# Food diversity: its relation to children’s health and consequent economic burden

**DOI:** 10.1186/s12889-024-18530-w

**Published:** 2024-04-24

**Authors:** Alfiah Hasanah, Bayu Kharisma, Sutyastie S. Remi, Asep Muhammad Adam, Adiatma Y.M. Siregar

**Affiliations:** Center for Economics and Development Studies, Department of Economics, Faculty of Economics and Business, Universitas Padjadjaran, Bandung, Indonesia

**Keywords:** I15, I10, I12, Dietary diversity, DDS, DSS, Children’s health, Economic burden, West Java

## Abstract

**Background:**

This study investigates the impact of low food diversity on the health status of children using the Dietary Diversity Score (DDS) and Dietary Serving Score (DSS) in a sub-district with the highest percentage of poor households. The economic burden of low food diversity was observed by analysing the cost of illness in the children with low food diversity.

**Methods:**

Data from 329 children were collected. We determined the impact of DDS and DSS and other factors on the health status of children aged 2–14 years, using a probit model. The cost of illness (e.g., typhus, stomach ulcers, coughs, flu, and fever) due to low food diversity was calculated from medical registration fees, medical action costs, transportation costs, and other costs.

**Results:**

The results shows that a 1% point increase in DDS or DSS potentially decreases children’s health complaints by 10% and 8%, respectively. Given the current 26% prevalence of health complaints among children with low DDS, the annual economic burden reaches US$75.72 per child per household. In addition, the current 41% prevalence of children with low DDS resulted in an annual cost to the government of US$153.45 per child.

**Conclusions:**

The effect of inadequate dietary diversity on children’s health is potentially high and contributes to the economic burden on households and the government.

## Introduction

Globally, nearly 200 million children under 5 years of age suffer from stunting, wasting, or both. At least 340 million children experience vitamin and mineral deficiencies, while around 40 million children under the age of 5 are overweight or obese [1]. The condition of stunting in Southern Asia and Sub-Saharan Africa as reported by WHO in 2022 is quite alarming at 30.5% and 31.3% respectively [2]. Countries affiliated with the World Health Assembly aim to reduce the stunting rate by 40% by 2025 through the Scaling Up Nutrition (SUN) program [3].

Indonesia has committed both nationally and globally to ensure children’s health, including achieving the Sustainable Development Agenda by 2030 [4]. However, the National Socioeconomic Survey (SUSENAS) in March 2022 showed that 28.81% of children aged 0–17 years had health complaints in the previous month that disrupted their daily activities. Compared with the previous year, there was a 4.13% increase in child health complaints in 2022. Child malnutrition is also a major issue in Indonesia, with consistently high rates of stunting [4]. The prevalence of stunting in Indonesia has decreased from 27.7% in 2019 to 21.6% in 2022. However, this proportion still falls short of WHO targets of less than 20% [4].

The main dietary nutritional problems affecting children’s health in developing countries are mostly caused by a lack of dietary diversity, with their diets mainly consisting of plant-based foods and limited intake of fruits and vegetables [5]. One of the dietary challenges for developing countries is a reliance on cereal staple foods with insufficient intake of animal protein, fruits, and vegetables [5, 6]. Dietary diversity and dietary quality themselves can be influenced by several factors, such as cultural traditions, social and economic status, food practices, financial allocations, and food choices [7]. To assess the quality of food diversity and adequacy, researchers mostly use the Dietary Diversity Score (DDS) [8], which is often interpreted as the main indicator of a quality diet [8]. In addition, Krebs-Smith et al. have tried to use the dietary serving score (DSS) to assess the quality of food diversity [9].

Indonesia uses two food consumption guides: (1) A rounded pyramid-like shape (*Tumpeng Gizi Seimbang*) which is meant to represent the principles of balanced nutrition (diverse food, food safety, physical activity, and weight monitoring), including five food groups and their recommended portions; and (2) a plate guide (*Piring Makanku, Porsi Sekali Makan*), which illustrates the recommended proportions of food groups to be consumed at each meal [10]. The Indonesia Demographic and Health Survey (IDHS) data between in 2012 and 2017 [11] on the dietary diversity of children at the age of 6–23 months shows that there is an increase in each food group, such as the diversity of grains, roots and tubers, and vegetables. Diversity also increased in the meat and egg food group, but in the legumes food group it declined by 3.4%. However, the consumption of protein from eggs and meat is still relatively low when compared with that of cereals. Overall, 60% of children had a balanced diet, while 72% were given the minimum amount of food appropriate for their age and 40% being provided the minimum acceptable diet [11], leaving the rest 60% of not receiving the minimum acceptable diet.

Low dietary diversity is related to diseases due to malnutrition, such as stunting, and other diseases such as dyslipidemia, cardiovascular risk, and even metabolic syndrome [12–14]. Children with varied food consumption will increase food intake and have good metabolism, and children with high dietary diversity are more likely to have good health [12].

Indeed, a study in West Java, Indonesia (the most populous province in Indonesia with around 50 million of population, almost 20% of the population of Indonesia) showed that having a more diversified diet increased the possibility of having normal nutritional status, while having inadequate dietary variety is most likely among the causes that led to experiencing stunting and/or become severely stunted [15]. Unfortunately, another study found that the nutritional variety, as well as the quality and quantity of diversity in West Java, Indonesia, is low, especially among vulnerable populations such as nursing women and small children [16, 17]. However, the studies have not directly addressed the implications of low food diversity on specific disease complaints and the economic losses caused by low food diversity.

Given the issues that we have elaborated, this study aims to address the following research questions. First, what is the correlation between food diversity and the health status of children? This research uses two methods in calculating food diversity of children, namely DDS (for quality of food diversity) and DSS (for quantity of food diversity). To our knowledge, comprehensive research on dietary diversity in developing countries using both the DDS and DSS and focusing on children’s health is still limited, if any. Second, what is the economic burden of low dietary diversity of children as incurred by parents and the government? Understanding such burden may create better awareness to the public and the government of the importance and severity of the issue. We expect our study is able to show and support the importance of the quality and quantity of food diversity in developing countries, and can be used as a basis of further policy development addressing this topic.

## Material and method

Our analysis consisted of three parts. First, we calculated the food diversity of children using both DDS and DSS methods. Second, we investigated the impact of the status of food diversity of children (using DDS and DSS) on the health of children with low DDS and DSS score using probit regression. Third, we calculated the economic burden of low dietary diversity of children as incurred by parents and the government. This study received ethical approval from the Universitas Padjadjaran, Bandung, Indonesia (1081/UN6.KEP/EC/2022).

### Study setting

This study was conducted in the Tamansari sub-district, Tasikmalaya City, West Java, with a population of 79,392 people. The Tamansari sub-district has the poorest households, 13.48% of the total population, among 10 sub-districts in Tasikmalaya City. Tasikmalaya is the city with the highest poverty percentage among other cities in West Java Province, reaching above 10% for three consecutives from 2021 to 2023 [18]. One of the issues that prompted this study to be conducted in Tasikmalaya City is that the stunting prevalence rate is above the provincial average [19]. However, the stunting rate is related to children’s diet and nutrition [4]. We therefore assume that Tasikmalaya City has a low level of food diversity.

### Data collection

We used primary collected data obtained through a survey in the Tamansari sub-district, Tasikmalaya City, with a population of 79,392 people. We collected data on six food groups consumed by children within the last 24 h. The six food groups are cereals, vegetables, fruits, whole grains, meat/fish/eggs, and milk/dairy products [8]. The number of children’s aged 2–14 years is amount to 19,044 in the place of study. At 5.5% margin of error, a total sample of at least 325 children’s should be obtained based on Slovin sampling methods. In the end, we surveyed the parents of 358 children respondents aged 2–14 years. After data cleaning, 329 respondents were included in our analysis. The survey asked questions related to sociodemographics, children’s health conditions in the past month, food groups consumed, and costs incurred by parents when their children were sick in the past month. Written informed consent was obtained from each participant after clarification of the study objectives and activities.

We then identified the status of food diversity of children’s using both DDS and DSS. DDS was chosen given the following considerations, firstly, is that indicators like MDD-C (for children) are recommended by the WHO for tracking and assessing the nutritional status of certain populations [20]. In which Breastmilk is one of the eight dietary categories included in the MDD-C, which covers infants from 2 to 24 months. However, the children in our research range in age from 2 to 14 years. We used DDS due to the applicability of DDS in diverse settings. Secondly, our study uses DDS approach to calculate dietary diversity using six food groups (See Table 1) [8]. The six food groups in DDS are in line with Indonesia food guide program which is a rounded pyramid-like shape (Tumpeng Gizi Seimbang) and refer to the Pedoman Gizi Seimbang (PGS) or Balanced Nutrition Guidelines through the principles of Beragam, Bergizi, Seimbang dan Aman (B2SA) or the Diverse, Nutritious, Balanced, and Safe menu.

**Table 1:** Scores for each food group in DDS measurement

Food group	Dietary diversity score
Cereal	1
Diary and Cheese	1
Meat/fish/egg	1
Nuts	1
Vegetable	1
Fruit	1
**Total**	**6**

*Source*: B2SA

We used DSS in measuring food diversity since DSS considers both quality and quantity of food serving. In our study we considered the number of meals as this is in line with the Indonesia Balanced Nutrition Guidelines/*Pedoman Gizi Seimbang* (PGS) that encourage consuming food groups with a set number of servings. The calculation refers to the portion recommendation for each food group that is given a certain score. The recommendations for meal portions refer to the *Pedoman Gizi Seimbang* (PGS) or Balanced Nutrition Guidelines through the principles of *Beragam, Bergizi, Seimbang dan Aman* (B2SA) or the Diverse, Nutritious, Balanced, and Safe menu [10]. The scoring method takes into account the same six major food categories as in the DDS calculation, with each food group receiving a maximum score of 20 (see Table 2). Cereal and fruit groups received a maximum of 5 points for each five recommended servings. The dairy & cheese and nuts groups received a maximum of 1 point for each one recommended serving, whereas one recommended serving of meat received a maximum of 2 points. Three recommended servings from the vegetable group received a maximum of 6 points.

**Table 2:** Scores and serving recommendations from each food group

Food group	Serving (B2SA)	Assigned score
Cereal	5	5
Diary and Cheese	1	1
Meat/fish/egg	1	2
Nuts	1	1
Vegetable	3	6
Fruit	5	5
**Total**		**20**

*Source*: B2SA

The children’s health status was identified based on the children health complaints within the last month, including typhus, stomach ulcers, coughs, flu, and fever.

### Regression analyses

We used two probit model regressions to identify the factors that cause children’s health complaints. The first model used DDS as an independent variable, while the second model used DSS. Variables used in the estimation method can be seen in Table 3. In general, the probit model equation can be represented by the following equation:

$$ {P}_{i}=f\left({Y}_{i}\right)$$

1 $$ =f\left({\beta }_{0}+{\beta }_{1}{X}_{1i}+\dots +{\beta }_{p}{X}_{pi}\right)$$

From the above equation, the function can be transformed into a linear form as follows:

2 $$ {f}^{-1}\left({P}_{i}\right)={\beta }_{0}+{\beta }_{k}{X}_{ki}+\dots +{\beta }_{p}{X}_{pi}$$

Where $$ {P}_{i}$$ is the probability of success in the i-th model, while $$ {f}^{-1}\left({P}_{i}\right)$$ is a normal cumulative distribution function (CDF), $$ {\beta }_{k}$$ and $$ {X}_{ki}$$ respectively are the coefficients of the regression model parameters and independent variables with k = 1,2, 3…, p.

**Table 3:** Variable description

Variable	Description	Unit
*Health Complaints*	Health complaints experienced by children with low food diversity (typhus, stomach ulcers, coughs, flu, and fever).	1 = Yes 0 = Otherwise
*DDS (Dietary Diversity Score)*	The food diversity score is divided into two parts, namely below average and above average	1 = Above the average (> 3.863222) 0 = Below the average (< 3.863222)
*DSS (Dietary Serving Score)*	The dietary serving score is divided into two parts, namely below and above average	1 = Above the average (> 16.74468) 0 = Below the average (< 16.74468)
*Food intake*	The frequency of meals the child eats in one day	Intervals ranging from 1 to 5
*Age child*	Age of children	Year
*Gender child*	Child gender	1 = Boy 0 = Girl
*Vaccine*	Completed COVID-19 vaccine	1 = Yes 0 = Otherwise
*Mother age*	Age of mother	year
*Housewife*	Mother’s employment status	1 = Work 0 = Housewife
*HH income*	Household income is divided into two parts, namely above and below average of all sample	1 = Above the average (> Rp. 2.290.237) 0 = Below the average (< Rp. 2.290.237)
*HH member*	Number of family members	Person
*Distance*	Distance from home to health care facility	1 = More than 1 km 0 = Otherwise

### Calculating economic burden

To answer the investigation number three, we analysed the economic burden of low food diversity. We included the illness experienced by children with low food diversity. The cost of child illness is calculated using direct medical costs and cost to the government. According to Nørgaard et al., direct costs can be calculated using medical expenses such as medical registration fees, medical action costs, transportation costs, and other costs [21]. We included these costs in our survey to obtain costs from the patients’ perspective. The calculation of direct costs in this study refers to the research of Rein et al. and represents medical costs and transfer fees from the government. The formula used in calculating direct medical costs (assuming one visit per month, 12 visits per year) is as follows [22]:

3 $$ Medical\, cost=Prevalence\, x\, Population\, x \,Direct \,Cost \text{x} 12$$

Prevalence is the proportion of children experiencing health complaints, population is the total number of respondents, and direct medical cost is the amount spent by parents when children experience health complaints. Meanwhile, the formula used to calculate the economic burden borne by the government due to low food diversity is as follows:

4 $$ Gov\_Cost=Prevalence x Population x Gov\_Assistance \text{x} 12$$

The economic costs borne by the government are calculated based on the prevalence of children who have low food diversity multiplied by the total number of respondents receiving assistance from the government. *Bantuan Sosial Non Tunai* (BSNT) or Non-Cash Social Assistance is used as an indicator of government assistance provided to the lower middle class, where the amount of assistance given if it is monetized is as much as $23.32 (Rp. 200,000) monthly, one aim of which is to improve child nutrition. We are using BSNT as a proxy for government costs because the aim of this program is to improve coverage of food and nutritional needs in the community. In addition, this program is one of the government’s efforts to alleviate stunting.

## Results

Table 4 summarizes the characteristics of the respondents, including those of the parents, households, and children.

**Table 4:** Summary statistics (*n* = 329)

Variables	Min	Max	Mean	Standard Deviation
Mothers age	20	53	35	5.85
Households income	1,000,000	30,000,000	2,293,313	2,950,760
Distance (1: > 1 km, 0: otherwise)	0	1	0.26	0.45
Households size	2	8	4.50	1.04
Health Complaints (1: yes, 0: otherwise)	0	1	0.25	0.43
Food intake	1	5	2.57	0.61
Child age	2	14	8.55	3.19
Child gender (1: boy, 0: girl)	0	1	0.51	0.50
Vaccine (1: completed, 0: otherwise)	0	1	0.01	0.10
Dietary Diversity Score (DDS) (1: above average, 0: below average)	0	1	0.58	0.49
Dietary Serving Score (DSS) (1: above average, 0: below average)	0	1	0.34	0.47

The average age of the mothers was 35 years, with an average household monthly income of 2.29 million rupiah. Around 70.82% of respondents lived more than one kilometre from a health-care facility. On average, respondents lived with five family members. More than 25% of children experienced health complaints during one month, and most children had three meals a day (52.89%). There are almost equal numbers of boys and girls in our sample. Around 98.78% of children did not complete their COVID-19 vaccination. Among all children in the study, 58.66% had below average DDS and around 34.95% had below average DSS.

Table 5 shows the result of probit regression of the DDS and DSS models. Using DDS as a proxy for dietary diversity, the first model shows that children with a high level of dietary diversity may not experience health complaints. Children with below average DDS and less frequent food intake are more likely to experience more health complaints.

**Table 5:** Estimation results of DDS and DSS model

Variables	(1)	(2)	(3)	(4)
	Health complaints^a^	Marginal effect^a^	Health complaints^b^	Marginal effect^b^
DDS (1 = above average)	-0.353***	-0.104		
	(0.165)	(0.048)		
DSS (1 = above average)			-0.302*	-0.089
			(0.172)	(0.050)
Food intake	-0.458**	-0.135	-0.408***	-0.120
	(0.152)	(0.043)	(0.160)	(0.046)
Age child	0.015***	0.004	0.025	0.007
	(0.027)	(0.008)	(0.266)	(0.008)
Gender child (1 = boy)	0.344**	0.101	0.367**	0.108
	(0.163)	(0.047)	(0.163)	(0.047)
Vaccine (1 = completed)	0.776	0.228	0.623	0.184
	(0.644)	(0.189)	(0.655)	(0.193)
Mothe rage	-0.010	-0.003	-0.008	-0.002
	0.015	(0.005)	(0.015)	(0.005)
Housewife (1 = work)	-0.100	-0.029	-0.108	-0.031
	(0.190)	(0.056)	(0.191)	(0.056)
HH income	0.490*	0.144	0.417**	0.123
	(0.189)	(0.055)	(0.186)	(0.054)
HH member	-0.041	-0.012	-0.043	-0.012
	(0.091)	(0.460)	(0.091)	(0.027)
Distance (1 = >1 km)	0.310*	0.091	0.323**	0.095
	(0.180)	(0.052)	(0.180)	(0.053)
Pseudo R^2^	0.087		0.083	
R-squared	329		329	

Robust standard errors in parentheses; *** *p* < 0.01, ** *p* < 0.05, * *p* < 0.1; ^a^Probit result of DDS model; ^b^Probit result of DSS model

As children grow older, they are more likely to experience a 0.4% point increase in health complaints (male children experience 10% point health complaints). Children from higher-income households have a 0.14% point, or 14%, higher probability of experiencing health complaints than children from lower-income households. Children live with a distance of more than 1 km from health facilities have a 0.012% point, or 1.2%, higher probability of experiencing health complaints compared with children who live closer to health facilities.

In the second model (Table 5, column 3), the relationship between dependent and independent variables remains consistent—with the exception of the *agechild* variable, which has an insignificant effect on health complaints. Children with lower DSS have a significantly higher odds ratio for experiencing health complaints than do children with higher DSS. Although the results of these models have similar signs and magnitudes, several differences appear, related to the probability. It is worth mentioning that there are statistically significant differences in estimates for food intake, the child’s sex, household income, and distance from a health care facility.

### Economic burden due to inadequate food diversity

As seen in Table 6, the average direct medical cost incurred by parents when their children have a health complaint is 98,696 rupiah (Rp) per visit. This cost represents 5% of the average income of households with low dietary diversity. Therefore, a household that have a child with low dietary diversity need to spend around 1,184,352 rupiah or $75.73 in to cover yearly direct medical cost (12 visits). Based on the 26% prevalence within the Tamansari sub-district, the overall direct medical cost amount to 100,669,920 rupiah or $6,436 per year (covering 85 households).

By using data on the incidence or probability of low food diversity in the Tamansari sub-district area and calculating the costs of the government program to prevent low diversity, the *Bantuan Sosial Non Tunai* (BSNT), government must bear the economic cost of 2,400,000 rupiah or $153.45 per child per year, or 326 million for the Tamansari sub-district in a year.

**Table 6:** Economic burden of individual and government^a^

Individual economic cost of health complaints due to low food diversity					
	Prevalence	Population	Medical cost average	Individual cost (Annual)	Economic cost
Health Complaint	26%	329	98,696 rupiah/$6.31	1,184,352 Rupiah/$75.73	100,669,920 Rupiah/$6,436
**Government Economic Cost/Spending for Low Food Diversity**					
	**Prevalence**	**Population**	**Non-Cash Social Assistance (BSNT)**	**Individual Cost (Annual)**	**Economic Cost**
Low Food Diversity*	41%	329	200,000 rupiah/$12.79	2,400,000 Rupiah/$153.45	326,400,000 rupiah/$20,869

* Total number of children with low dietary diversity, ^a^)we use exchange rate USD to IDR in January 2023

## Discussion

Our study has established the correlation between the quality and quantity of dietary diversity on the children health complains. We have also shown the subsequent economic burden per children. Based on these findings, the following observations prevail.

First, our estimation results found that lower DDS and DSS score may potentially result in more children health complaints. The DDS has been shown to be favourably associated with nutritional adequacy, including appropriate macro- and micronutrient consumption, particularly in children [23]. However, the relationship between DDS and certain children health issues is not widely discussed in a previous study and emphasized on the overall relationship between DDS and nutritional status [24]. Despite the different contexts, the results we found has the same direction, with dietary diversity contributing negatively to children’s health complaints [25]. In terms of quantity (DSS), we found that the closer a child is the prescribed meal portions (*Pedoman Gizi Seimbang*/Balanced Nutrition Guidelines), the better the health of the child. Lee & Ryu stated that a person with a low level of food diversity is more susceptible to disease [25]. Eating frequency has an effect on reducing obesity and other health problems in children, such as typhoid and diarrhoea [26–30].

These results provide the basis for designing the proper policy to support better quality and quantity of dietary diversity. Fortunately, Indonesia has long promoted such concept using the previously mentioned food guide program (a rounded pyramid-like shape) and the balanced nutrition guidelines. However, the implementation of the program and guideline still remains a challenge as pointed out by other studies in West Java provinces showing the low quantity and quality of dietary diversity [16, 17], requiring more active promotion interventions. An approach adopted by Lao People’s Democratic Republic (Lao PDR) can be considered, especially in a resource limited context. The approach has managed to improve child dietary diversity through educational approaches to caregivers of children aged 6–59 months and utilizing locally available food [31]. The intervention also include maternal and child health services, educational and awareness activities related to food and nutrition, and cooking demonstrations. As Indonesia already has the monthly maternal and child health services conducted at the community level at the integrated healthcare post (*posyandu*) which includes education on nutrition [32], incorporating further emphasize to utilize local food and provide additional intervention such as providing cooking demonstration may provide more positive results. Another approach is to utilize or enhance the agroecological practices. A study shows that some practices such as crop diversification, agroforestry, mixed crop and livestock systems, and improving soil quality is estimated to have positive outcomes on dietary diversity [33]. As agriculture is one of a key sector in Indonesia, this approach can potentially benefit the agriculture sector and improve dietary diversity (quality and quantity) and health outcome. Future studies can further explore this approach within the Indonesian context.

Second, our study has shown that the estimated annual direct medical cost to treat health complaints of children who has low DDS and DSS score amount to US$75 per child. By adjusting exchange rate to USD 2023, previous studies found that the annual cost of child health complaints across low- and middle-income countries was $31.15 [34]. Meanwhile, in developing countries such as Bangladesh, a comparison of several studies conducted found that the direct costs incurred due to health complaints were $0.79 [35], $3.49 [36], and $21.23 [37] in USD 2023 respectively. Direct costs for health complaints in India range from $1.71 [38] to $21.27 [39]. While these variation in costs may be due to the different methods used across the literatures, our estimation falls at the higher range. This further supports the importance of increasing both the quality and quantity of food diversity to be consumed by children in order to avoid such cost.

In addition, it appears that a child’s age, sex, income, and distance to health care all have an impact on their health complaints. According to Dafursa & Gebremedhin, the level of food diversity in children increases with age: children approaching adolescence have diets with greater food diversity than do younger children [40]. In this study, our sample is the sub-district with the highest poverty rate in Tasikmalaya City, where we also found in our sample data that 73.25% of household incomes are below average. We do not expect that high parental income will reduce the possibility of children experiencing health complaints. This is indicated by the results of household income estimates, which are positively correlated with health complaints. Currie & Goodman have stated that income as a measure of socioeconomic status (SES) has a relatively small effect on children’s health [41].

This study has some limitations. First, the data coverage, treatment, and population characteristics in each region may be different. To be able to fully describe the condition of food diversity and the economic impact caused by low food diversity, data must cover a wider scale, such as at the city, district, or even provincial level. Thus, our results should be generalized with caution. However, our results do provide a snapshot of the situation in a poorer area in Indonesia. Second, the diseases experienced by children may not be caused by low food diversity. However, we tried to be careful to isolate the diseases by selecting the characteristics of children who experience health complaints and have low food diversity.

## Conclusion

This study investigates the impact of low dietary diversity on the health problems of children in the sub-district with the highest level of poverty in Tasikmalaya City. We used DDS and DSS as indicators to measure dietary diversity in children. We found that food diversity in Tasikmalaya City, as measured by the DDS, was relatively low. Around 58.66% of children had food diversity below average. Our results show that the lower the food diversity, the more likely it is that children have health problems that potentially lead to a relatively high economic burden. The economic cost of health problems due to lower dietary diversity takes almost 5% of the average income of households with low dietary diversity in the sub-district where the poverty level above the average in Tasikmalaya city. Policies to improve the quality and quantity of dietary diversity is urgently required, especially in the poorer area in a resource limited-setting country.

## Data Availability

The dataset used in this study is available from the corresponding author upon reasonable request.

## Abbreviations

DDS: Dietary Diversity Score
DSS: Dietary Serving Score
SUN: Scaling Up Nutrition
MDD-7: Minimum Dietary Diversity
CDF: Cumulative Distribution Function
PGS: *Pedoman Gizi Seimbang* (Balanced Nutrition Guidelines)
B2SA: *Bergizi, Seimbang dan Aman* (Nutritious, Balanced, and Safe)
BSNT: *Bantuan Sosial Non Tunai* (Non-Cash Social Assistance
SES: Socioeconomic Status
IDHS: Indonesia Demographic and Health Survey

## References

[1] UNICEF. (2019) Children, food and nutrition: growing well in a changing world.

[2] WHO. (2022) Stunting prevalence among children under 5 years of age (%) (model-based estimates). https://www.who.int/data/gho/data/indicators/indicator-details/GHO/gho-jme-stunting-prevalence. Accessed 10 Feb 2024.

[3] UNICEF. (2013) Improving child nutrition: the achievable imperative for global progress.

[4] UNICEF. (2020) The State of Children in 2020 Indonesia.

[5] Arimond M, Ruel MT (2004). Dietary diversity is Associated with Child Nutritional Status: evidence from 11 demographic and health surveys. J Nutr.

[6] Ochola S, Masibo PKinya (2014). Dietary intake of schoolchildren and adolescents in developing countries. Ann Nutr Metab.

[7] WHO. (2006) SEA-NUT-163 distribution: General Adolescent Nutrition: a review of the Situation in selected South-East Asian Countries ii Adolescent Nutrition: a review of the Situation in selected South-East Asian countries.

[8] Rathnayake KM, Madushani P, Silva K (2012). Use of dietary diversity score as a proxy indicator of nutrient adequacy of rural elderly people in Sri Lanka. BMC Res Notes.

[9] Krebs-Smith SM, Smiciklas-Wright H, Guthrie HA, Krebs-Smith J (1987). The effects of variety in food choices on dietary quality. J Am Diet Assoc.

[10] Minister of Health Regulation (PMK). (2014) Pedoman Gizi Seimbang. Jakarta.

[11] National Population and Family Planning Board, Indonesia S. Ministry of Health (2017) Indonesia Demographic and Health Survey 2017.

[12] Frempong RB, Annim SK (2017). Dietary diversity and child malnutrition in Ghana. Heliyon.

[13] Gassara G, Chen J (2021). Household food insecurity, dietary diversity, and stunting in sub-saharan africa: a systematic review. Nutrients.

[14] Berhane HY, Jirström M, Abdelmenan S, Berhane Y, Alsanius B, Trenholm J, Ekström EC. (2020) Social Stratification, Diet Diversity and Malnutrition among Preschoolers: A Survey of Addis Ababa, Ethiopia. Nutrients 2020, Vol 12, Page 712 12:712. 10.3390/nu12030712 PMC7146462 32156006

[15] Rahmannia S, Diana A, Luftimas DE, Gurnida DA, Herawati DMD, Houghton LA, Gibson RS (2019). Poor dietary diversity and low adequacy of micronutrient intakes among rural Indonesian lactating women from Sumedang district, West Java. PLoS ONE.

[16] Agustina R, Nadiya K, El Andini A, Setianingsih AA, Sadariskar AA, Prafiantini E, Wirawan F, Karyadi E, Raut MK (2020). Associations of meal patterning, dietary quality and diversity with anemia and overweight-obesity among Indonesian school-going adolescent girls in West Java. PLoS ONE.

[17] Sekiyama M, Tsuchiya K, Matsuda H. (2023) Socioecological and dietary change from 2001 to 2015 in rural West Java, Indonesia. 10.21203/RS.3.RS-2642208/V1.

[18] Statistical Center of West Java Province. (2023) Percentage of Poor Population (Percent), 2021–2023. https://jabar.bps.go.id/indicator/23/51/1/persentase-penduduk-miskin.html. Accessed 29 Feb 2024.

[19] Open Data Jawa Barat. (2023) Prevalence of stunting in West Java decreased by 4.3%, achieving WHO target is getting closer. https://opendata.jabarprov.go.id/id/artikel/data-terbaru-prevalensi-stunting-di-jabar-menurun-43-pencapaian-target-who-semakin-dekat. Accessed 29 Feb 2024.

[20] WHO, UNICEF. (2017) Global Nutrition Monitoring Framework Operational Guidance for tracking Progress in Meeting Targets For 2025.

[21] Nørgaard SK, Vissing NH, Chawes BL, Bisgaard H, Stokholm J, Bønnelykke K. (2021) Cost of Illness in Young Children: A Prospective Birth Cohort Study. Children. 10.3390/CHILDREN8030173. 10.3390/children8030173 PMC7996350 33668336

[22] Rein DB, Wittenborn JS, Zhang P, Sublett F, Lamuda PA, Lundeen EA, Saaddine J (2022). The Economic Burden of Vision loss and blindness in the United States. Ophthalmology.

[23] Yari Z, Amini M, Rasekhi H, Nikooyeh B, Doustmohammadian A, Ghodsi D, Rabiei S, Neyestani TR (2022). Dietary diversity and its relationship with nutritional adequacy in 24 to 59 months old children in Iran: study protocol. BMC Nutr.

[24] Zhao W, Yu K, Tan S, Zheng Y, Zhao A, Wang P, Zhang Y (2017). Dietary diversity scores: an indicator of micronutrient inadequacy instead of obesity for Chinese children. BMC Public Health.

[25] Lee SJ, Ryu HK (2018). Relationship between dietary intakes and the double burden of malnutrition in adults of Malang, Indonesia: an exploratory study. Nutr Res Pract.

[26] Ruidavets JB, Bongard V, Bataille V, Gourdy P, Ferrières J. (2002) Eating frequency and body fatness in middle-aged men. International Journal of Obesity 2002 26:11 26:1476–1483. 10.1038/sj.ijo.0802143 12439650

[27] Ma Y, Bertone ER, Stanek EJ, Reed GW, Hebert JR, Cohen NL, Merriam PA, Ockene IS (2003). Association between Eating Patterns and Obesity in a free-living US Adult Population. Am J Epidemiol.

[28] Yannakoulia M, Melistas L, Solomou E, Yiannakouris N (2007). Association of Eating Frequency with body fatness in pre- and Postmenopausal women. Obesity.

[29] Drummond SE, Crombie NE, Cursiter MC, Kirk TR. (1998) Evidence that eating frequency is inversely related to body weight status in male, but not female, non-obese adults reporting valid dietary intakes. International Journal of Obesity 1998 22:2 22:105–112. 10.1038/sj.ijo.0800552 9504318

[30] Duval K, Strychar I, Cyr MJ, Prud’homme D, Rabasa-Lhoret R, Doucet É (2008). Physical activity is a confounding factor of the relation between eating frequency and body composition. Am J Clin Nutr.

[31] Sato Y, Khamphithoun S, Saiyachak K, Ando H, Ishizuka T, Saeki S, Miyoshi M. (2024) Enhancing child dietary diversity through cooking demonstration and nutritional education in rural Lao PDR. Tropical Medicine and Health 2024 52:1 52:1–15. 10.1186/s41182-023-00571-3 PMC10773088 38191472

[32] Ministry of Health of the Republic of Indonesia. (2018) The importance of integrated healthcare post for children. https://promkes.kemkes.go.id/?p=8960. Accessed 29 Feb 2024.

[33] Bezner Kerr R, Madsen S, Stüber M, Liebert J, Enloe S, Borghino N, Parros P, Mutyambai DM, Prudhon M, Wezel A (2021). Can agroecology improve food security and nutrition? A review. Glob Food Sec.

[34] Baral R, Nonvignon J, Debellut F, Agyemang SA, Clark A, Pecenka C (2020). Cost of illness for childhood diarrhea in low- and middle-income countries: a systematic review of evidence and modelled estimates. BMC Public Health.

[35] Rheingans R, Kukla M, Faruque ASG, Sur D, Zaidi AKM, Nasrin D, Farag TH, Levine MM, Kotloff KL (2012). Determinants of Household costs Associated with Childhood Diarrhea in 3 south Asian settings. Clin Infect Dis.

[36] Das J, Das SK, Ahmed S (2015). Determinants of percent expenditure of household income due to childhood diarrhoea in rural Bangladesh. Epidemiol Infect.

[37] Sarker AR, Sultana M, Mahumud RA (2018). Economic costs of hospitalized diarrheal disease in Bangladesh: a societal perspective. Glob Health Res Policy.

[38] Mendelsohn AS, Asirvatham JR, Mkaya Mwamburi D, Sowmynarayanan TV, Malik V, Muliyil J, Kang G (2008). Estimates of the economic burden of rotavirus-associated and all-cause diarrhoea in Vellore, India. Tropical Med Int Health.

[39] Mathew A, Srinivasan R, Venugopal S, Kang G (2016). Direct medical costs in children with rotavirus and non-rotavirus diarrhea admitted to a pediatric intensive care unit and high dependency unit in Delhi. Indian Pediatr.

[40] Dafursa K, Gebremedhin S. Dietary diversity among children aged 6–23 months in Aleta Wondo District. Southern Ethiopia; 2019. 10.1155/2019/2869424 PMC6878804 31815015

[41] Currie J, Goodman J. (2020) Parental socioeconomic status, child health, and human capital. The Economics of Education: A Comprehensive Overview 239–248.

